# Functional Characterization of the *Paeonia ostii P5CS* Gene under Drought Stress

**DOI:** 10.3390/plants13152145

**Published:** 2024-08-02

**Authors:** Yuting Luan, Honglei An, Zijie Chen, Daqiu Zhao, Jun Tao

**Affiliations:** 1 College of Horticulture and Landscape Architecture, Yangzhou University, Yangzhou 225009, China; luanyuting96@gmail.com (Y.L.); 13961828931@163.com (H.A.); taomi34215708@163.com (Z.C.); dqzhao@yzu.edu.cn (D.Z.); 2 Joint International Research Laboratory of Agriculture and Agri-Product Safety, The Ministry of Education of China, Yangzhou University, Yangzhou 225009, China

**Keywords:** *Paeonia ostii*, *P5CS*, proline, drought stress

## Abstract

With persistent elevation in global temperature, water scarcity becomes a major threat to plant growth and development, yield security, agricultural sustainability, and food production. Proline, as a key osmolyte and antioxidant, plays a critical role in regulating drought tolerance in plants, especially its key biosynthetic enzyme, delta-1-pyrroline-5-carboxylate synthase (P5CS), which always positively responds to drought stress. As an important woody oil crop, the expansion of *Paeonia ostii* cultivation needs to address the issue of plant drought tolerance. Here, we isolated a *PoP5CS* gene from *P. ostii*, with an open reading frame of 1842 bp encoding 613 amino acids. *PoP5CS* expression progressively increased in response to increasing drought stress, and it was localized in the cytoplasm. Silencing of *PoP5CS* in *P. ostii* reduced drought tolerance, accompanied by decreased proline content, elevated reactive oxygen species (ROS) accumulation, and increased relative electrical conductivity (REC) and malondialdehyde (MDA) levels. Conversely, overexpression of *PoP5CS* in *Nicotiana tabacum* plants enhanced drought resistance, manifested by increased proline levels, reduced ROS accumulation, and lower REC and MDA contents. This study isolates *PoP5CS* from *P. ostii* and validates its role in regulating drought tolerance, providing valuable genetic resources and theoretical insights for the development of drought-resistant *P. ostii* cultivars.

## 1. Introduction

In the face of a warming planet and increasingly unpredictable weather patterns, drought has emerged as a pressing global threat to agricultural productivity and ecosystem stability. As a primary environmental stressor, drought significantly impacts plant growth and development, often leading to reduced yield and even plant death [1]. To survive and thrive under these adverse conditions, plants have evolved intricate mechanisms of osmotic adjustment, which play a pivotal role in mitigating the deleterious effects of drought stress. Osmotic adjustment is a physiological response that involves the accumulation of compatible solutes, such as proline, glycine betaine, and soluble sugars, within plant cells to maintain turgor pressure and cellular homeostasis [2,3,4,5,6]. Recent studies have underscored the complexity and versatility of these mechanisms, revealing that different plant species and even cultivars within the same species exhibit distinct patterns of coping abilities and osmotic adjustment in response to drought [7,8].

Among the various adaptive mechanisms employed by plants to mitigate the detrimental effects of drought, the accumulation of proline stands out as a pivotal response. Proline, a cyclic subamino acid, has garnered considerable attention in recent years for its multifaceted roles in conferring drought tolerance to plants. Its accumulation under drought stress is not merely a passive consequence of cellular dehydration but an active adaptive strategy that serves to stabilize subcellular structures, maintain osmotic balance, and scavenge reactive oxygen species (ROS) generated during stress periods [9,10]. Proline biosynthesis is primarily mediated by the enzymes delta-1-pyrroline-5-carboxylate synthase (P5CS) and pyrroline-5-carboxylate reductase (P5CR), while its degradation is catalyzed by proline dehydrogenase (ProDH) and pyrroline-5-carboxylate dehydrogenase (P5CDH). This metabolic flux is tightly regulated in response to drought, with increased P5CS activity and suppressed ProDH activity leading to enhanced proline accumulation [11]. The functions of proline and the *P5CS* gene in plant responses to abiotic stresses have been explored in multiple species. As a key rate-limiting enzyme for proline biosynthesis, the *P5CS* gene was first isolated from *Vigna aconifolia* in 1992 by Hu et al. [12]. Since then, a series of plant *P5CS* genes have been cloned and their functions have received widespread attention. In the model plant *Arabidopsis thaliana*, P5CS protein is encoded by two differentially regulated genes, *AtP5CS1* and *AtP5CS2*. The abundance of *AtP5CS* transcripts is tissue-specific and could be induced by drought, salinity, or ABA [13]. Zhang et al. [14] isolated the promoter of the *A. thaliana P5CS* gene and analyzed the effect of NaCl stress on *P5CS* promoter activity and found that NaCl stress induced an increase in P5CS promoter activity. Yamchi et al. [15] generated multiple *A. thaliana P5CS* gene overexpression lines and found that transgenic plants have higher proline expression levels and greater resistance to osmotic stress compared to non-transgenic plants. In addition to *A. thaliana*, the potential of the *P5CS* gene to respond to stress has also been reported in other species. For, example, in *Oryza sativa*, the *OsP5CS1* gene’s expression was induced by a wide range of abiotic stresses (NaCl, drought, low temperature) and was commonly expressed in a variety of organs, whereas *OsP5CS2* was specifically highly expressed in mature plants and induced by NaCl and mannitol [16]. *Medicago truncatula MtP5CS2* has a positive role in osmotic regulation [17]. In Oriental hybrid lily Sorbonne, the expression of *LhSorP5CS* was upregulated by NaCl, mannitol, and ABA and accompanied by more proline accumulation [18]. Therefore, studying proline accumulation under drought stress and the function of the *P5CS* gene could provide valuable insights into how plants adapt to and survive under water deficit conditions.

*Paeonia ostii*, originating from China, is a perennial woody oil crop with ornamental, medicinal, and economic values [19]. Its seed oil boasts a high content of unsaturated fatty acids up to 92%, including 42% of α-linolenic acid, making it popular in the market due to its health benefits. Given the scarcity of arable land in China, *P. ostii* is predominantly cultivated in arid regions such as hills and slopes, where water scarcity significantly affects its growth, development, and yield [20]. For further expansion of *P. ostii’s* area for cultivation in arid and semi-arid regions, previous scholars have worked on finding ways to alleviate drought damage in *P. ostii*. Several cultivation practices including exogenous calcium treatment, silicon treatment, ferulic acid treatment, graphene oxide treatment, and fulvic acid treatment have been utilized to enhance the mitigation of this stress [19,21,22,23,24]. At the level of drought-tolerant gene identification, potential drought-tolerant structural genes (*CCoAOMT*, *LACS4*) and the transcription factor STAT were unearthed and were thought to be involved in drought tolerance in *P. ostii* [25,26,27]. However, the relationship between the *P5CS* gene and drought tolerance in *P. ostii* has not been reported. Here, a *P5CS* gene positively responding to drought stress was identified in *P. ostii*. Through gene cloning, expression pattern analysis, subcellular localization observation, and virus-induced *PoP5CS* gene silencing and overexpression experiments, we explored the positive role of the *PoP5CS* gene in regulating drought tolerance in *P. ostii*. This is the first characterization of the *PoP5CS* gene’s function in *P. ostii* drought regulation, which contributes to our understanding of the molecular mechanism of proline-regulated drought tolerance in *P. ostii*.

## 2. Results

### 2.1. PoP5CS Positively Responds to Drought Stress

**Figure 1:**
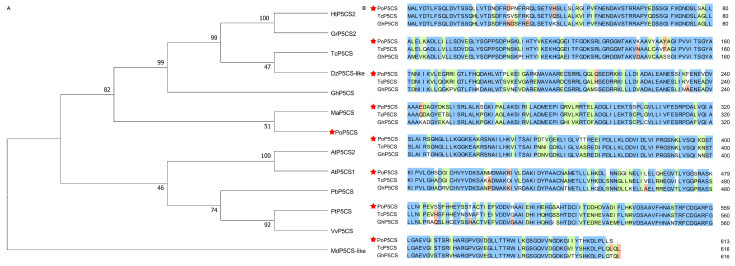
Sequences analysis of *PoP5CS*. (**A**) A phylogenetic tree constructed using PoP5CS and P5CS proteins from other plant species. TcP5CS (*Theobroma cacao*, XP_007026912.2), DzP5CS-like (*Durio zibethinus*, XP_022766207.1), HtP5CS2 (*Hibiscus trionum*, GMJ03143.1), GhP5CS (*Gossypium hirsutum*, XP_040936303.1), GrP5CS2 (*Gossypium raimondii*, XP_012458611.1), AtP5CS1 (*Arabidopsis thaliana*, NP_001189714.1), AtP5CS2 (*A. thaliana*, NP_191120.2), MaP5CS (*Melia azedarach*, KAJ4704590.1), PtP5CS (*Populus trichocarpa*, XP_002315202.1), MdP5CS-like (*Malus domestica*, XP_008387828.2), VvP5CS (*Vitis vinifera*, XP_010658318.1), PbP5CS (*Pyrus × bretschneideri*, XP_009343959.2). PoP5CS is marked with a red pentagram. The neighbor-joining tree was generated by MEGA7.0 using a p-distance model with 1000 bootstrap replicates. The tree is a bootstrap consensus tree, and bootstrap values are shown at the nodes. (**B**) Multiple sequence alignment of PoP5CS and homologous proteins from other species. PoP5CS is marked with a red pentagram. The colors in the sequence alignment mean different homology levels. Orange means homology level ≥ 33%, green means homology level ≥ 50%, and blue means homology level = 100%.

**Figure 2:**
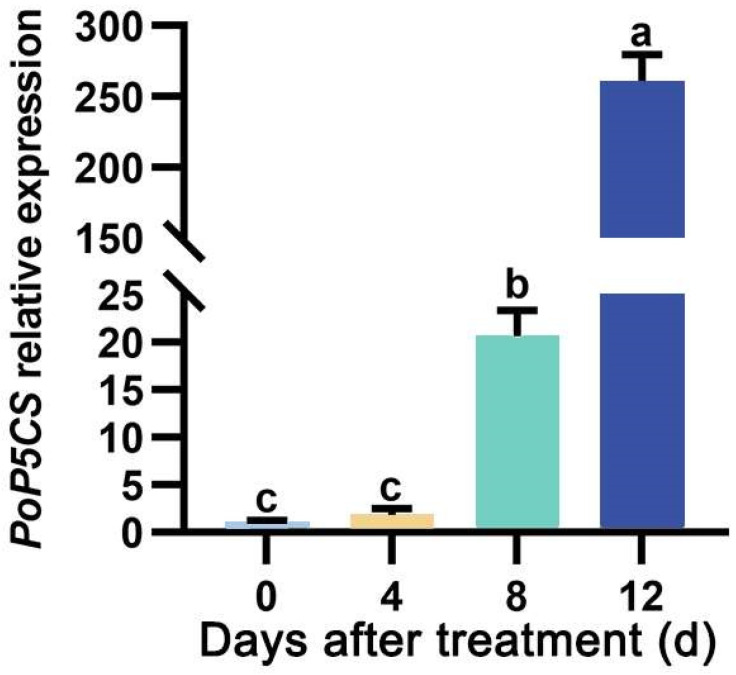
RT-qPCR analysis of the expression levels of *PoP5CS* under drought stress conditions on Days 0, 4, 8, and 12. *PoP5CS* expression on Day 0 is normalized to 1. Data represent the mean ± SD of three biological replicates, and letters indicate significant differences by one-way ANOVA (*p* < 0.05).

Based on a drought-related transcriptome database of *P. ostii* [28], we worked to find drought-responsive genes that regulate drought tolerance in *P. ostii*. We focused on the key osmotic regulation-associated proline biosynthesis gene, *P5CS*. We found a total of three differential *P5CS* genes (Unigene0006017, Unigene0000731, and Unigene0008549) (Table S1). Among them, Unigene0008549 had the highest FPKM value and the highest differential fold of 4.04, suggesting its positive response to drought stress. Then, the full length of Unigene0008549 was obtained by PCR, and its coding sequence was 1842 bp in length, encoding 613 amino acids. To resolve the evolutionary relationship between Unigene0008549 and other homologs, we downloaded eight P5CS proteins from the NCBI database and applied them to an evolutionary tree analysis with Unigene0008549. As shown in Figure 1A, Unigene0008549 clustered with *A. thaliana* AtP5CS1 and AtP5CS2 and shared 57% homology with them, indicating that Unigene0008549 belonged to the P5CS family, therefore named as PoP5CS. Multiple sequence comparison showed that there existed a high sequence similarity between PoP5CS and P5CS family members (Figure 1B). Subsequently, the expression profiles of *PoP5CS* in *P. ostii* leaves sampled on Days 0, 4, 8, and 12 after drought treatment were scrupulously examined by quantitative real-time PCR (RT-qPCR) analysis. As shown in Figure 2, the expression level of *PoP5CS* showed a continuous increase, which was 1.82 times higher than that of Day 0 on Day 4, 20.61 times higher than that of Day 0 on Day 8, and reached an astonishing multiplicity of 260.37 on Day 12. The above results were consistent with its upregulated expression pattern in the transcriptome database, which was further evidence of the importance of *PoP5CS* for *P. ostii* to respond to drought stress. The above results revealed that *PoP5CS* positively responded to drought stress.

### 2.2. PoP5CS is Located in the Cytoplasm

**Figure 3:**
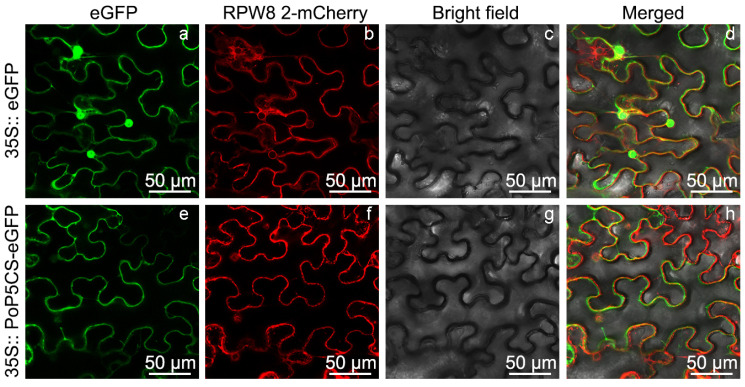
Subcellular localization of PoP5CS in *Nicotiana benthamiana* leaves. (**a**,**e**) The green fluorescence signals of the 35S:: eGFP expression vector and 35S:: PoP5CS-eGFP expression vector at 488 nm; (**b**,**f**) the red fluorescence signals of the RPW8 2-mCherry expression vector at 561 nm; (**c**,**g**) bright field images; (**d**) an overlaid image of (**a**–**c**); (**h**) an overlaid image of (**e**–**g**).

In order to further reveal the expression characteristics of PoP5CS protein, we utilized *Nicotiana benthamiana* leaves for subcellular localization. We transiently transformed fusion vectors containing P5CS protein and enhanced green fluorescent protein (eGFP) into *N. benthamiana* leaves. The fusion vector expressed in the *A. thaliana RPW8 2* gene (accession number: NM_114906.2) and mCherry fusion protein was used to indicate cytoplasmic localization signals. Following infiltration, fluorescent signals at 488 nm and 561 nm were elicited in *N. benthamiana* leaves to clarify the protein localization of PoP5CS. The emission of the eGFP signal, originating from the 35 S promoter-driven fusion construct of PoP5CS-eGFP, was observed within the cytoplasmic compartment of *N. benthamiana* leaves, where it co-localized with a cytoplasmic marker protein (Figure 3). This finding provided evidence that the subcellular localization of the PoP5CS protein occurs specifically within the cytoplasm of *N. benthamiana* leaves.

### 2.3. PoP5CS-Silenced P. ostii Show More Sensitivity to Drought

**Figure 4:**
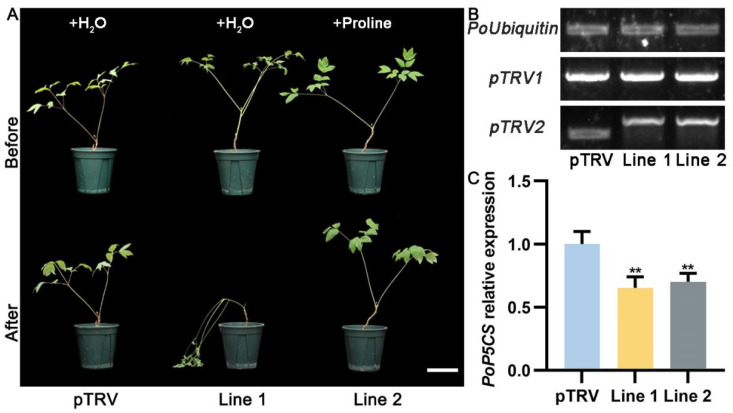
Phenotypic observation and positive plant identification of *PoP5CS*-silenced *Paeonia ostii*. (**A**) Phenotypic observation of *PoP5CS*-silenced *P. ostii* when *PoP5CS* was silenced. Bar = 10 cm. (**B**) PCR validation of *PoP5CS*-silenced *P. ostii* after 15 days of drought treatment. (**C**) RT-qPCR validation of *PoP5CS*-silenced *P. ostii* after 15 days of drought treatment. Data represent the mean ± SD of three replicates, and statistical significance was determined by Student’s *t*-test (** *p* < 0.01).

**Figure 5:**
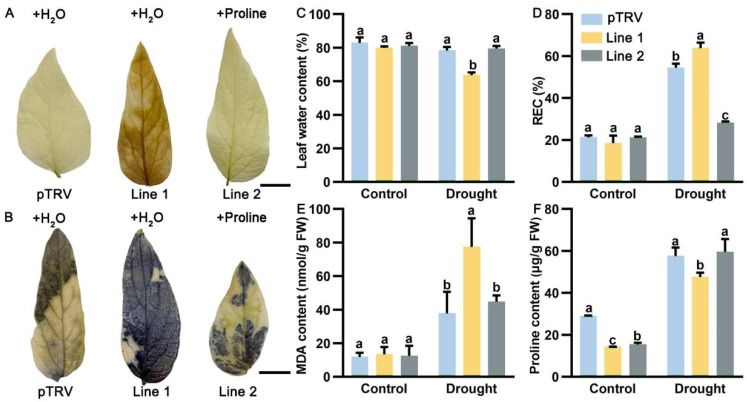
Physiological index measurement of *PoP5CS*-silenced *Paeonia ostii*. (**A**) H_2_O_2_ accumulation condition of *PoP5CS*-silenced *P. ostii* leaves after 15 days of drought treatment. Bar = 1 cm. (**B**) O_2_^−^ accumulation condition of *PoP5CS*-silenced *P. ostii* leaves after 15 days of drought treatment. Bar = 1 cm. (**C**) Leaf water content when *PoP5CS* was silenced. (**D**) REC when *PoP5CS* was silenced. REC, relative electrical conductivity. (**E**) MDA content when *PoP5CS* was silenced. MDA, malondialdehyde. (**F**) Proline content when *PoP5CS* was silenced. Data represent the mean ± SD of three replicates, and letters indicated significant differences by one-way ANOVA (*p* < 0.05).

To investigate the function of *PoP5CS* in regulating drought tolerance, we obtained control plants (*P. ostii* transformed with pTRV), as well as *PoP5CS*-silenced *P. ostii* line 1 and line 2 based on the virus-induced gene silencing (VIGS) technique, and cultured them to the leaf expanding stage. Before drought treatment, there was no significant difference in plant phenotypes among these plants (Figure 4A). Prior to the drought treatment, we treated *PoP5CS*-silenced *P. ostii* line 2 with foliar proline spray for 3 days, and control plants (*P. ostii* transformed with pTRV) and *PoP5CS*-silenced *P. ostii* line 1 were sprayed with deionized water as controls. Next, the above *P. ostii* plants were subjected to drought treatment. DNA and total RNA were extracted from control plants (*P. ostii* transformed with pTRV) and *PoP5CS*-silenced *P. ostii* line 1 and line 2 on Day 15 after drought treatment for positive transgenic plant validation. As shown in Figure 4B, when DNA was amplified using *P. ostii Ubiquitin-*specific primers and pTRV1-specific primers, all experimental plants had a bright band, whereas when pTRV2-specific primers were used to amplify DNA, the pTRV band position differed in length from *PoP5CS*-silenced *P. ostii* line 1 and line 2. In addition, we quantified the expression level of *PoP5CS* in the above plants and found that the expression level of *PoP5CS* in *PoP5CS*-silenced *P. ostii* line 1 and line 2 decreased compared with pTRV by 35.45% and 30.23%. When they were exposed to drought treatment, the control plants (*P. ostii* transformed with pTRV) and proline-treated *PoP5CS*-silenced *P. ostii* line 2 maintained a normal growth condition and did not show a drought damage phenotype (Figure 4A). In contrast, *PoP5CS*-silenced *P. ostii* line 1 plants showed drooping stems and wilted leaves, which might be attributed to the suppressed expression of *PoP5CS* (Figure 4A). Then, several physiological experiments were applied to investigate the deeper response of *PoP5CS* to drought. As shown in Figure 5A,B, H_2_O_2_ accumulation identified by diaminobenzidine (DAB) staining and O_2_^·−^ accumulation identified by nitrotetrazolium blue chloride (NBT) staining were used to assess the extent of ROS accumulation in *P. ostii* leaves. Among them, *PoP5CS*-silenced *P. ostii* line 1 plants showed large areas of brown and blue coloration in the leaves, implying a severe degree of drought damage. In contrast, control plants (*P. ostii* transformed with pTRV) and *PoP5CS*-silenced *P. ostii* line 2 plants showed little or almost no coloration in their leaves. Moreover, leaf water content, relative electrical conductivity (REC), and malondialdehyde (MDA) content all showed low levels under normal conditions (Figure 5C–E). When they were exposed to drought, *PoP5CS*-silenced *P. ostii* line 1 exhibited a more substantial decrease in water content, while control plants (*P. ostii* transformed with pTRV) and *PoP5CS*-silenced *P. ostii* line 2 showed a smaller decrease (Figure 5C). REC and MDA contents increased to a high extent in *PoP5CS*-silenced *P. ostii* line 1 and to a lesser extent in control plants (*P. ostii* transformed with pTRV) and *PoP5CS*-silenced *P. ostii* line 2 (Figure 5D,E). The above results suggest that *PoP5CS* played a positive role in regulating drought tolerance in *P. ostii*.

### 2.4. Overexpression of PoP5CS Increases Drought Tolerance in Nicotiana tabacum

**Figure 6:**
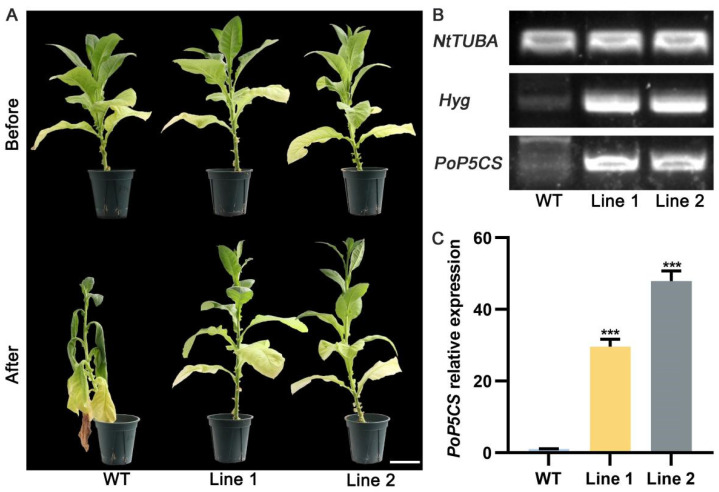
Phenotypic observation and positive plant identification of *PoP5CS*-overexpressing *Nicotiana tabacum*. (**A**) Phenotypic observation of *PoP5CS*-overexpressing *N. tabacum* when *PoP5CS* was overexpressed. Bar = 10 cm. (**B**) PCR validation of *PoP5CS*-overexpressing *N. tabacum* after 10 days of drought treatment. *NtTUBA*, specific primers in *N. tabacum* tubulin alpha chain coding sequence; *Hyg*, specific primers in the *hygromycin* label region of pCAMBIA1301 vector; *PoP5CS*, specific primers in *PoP5CS* coding sequence. (**C**) RT-qPCR validation of *PoP5CS*-overexpressing *N. tabacum* after 10 days of drought treatment. Data represent the mean ± SD of three replicates, and statistical significance was determined by Student’s *t*-test (*** *p* < 0.001).

**Figure 7:**
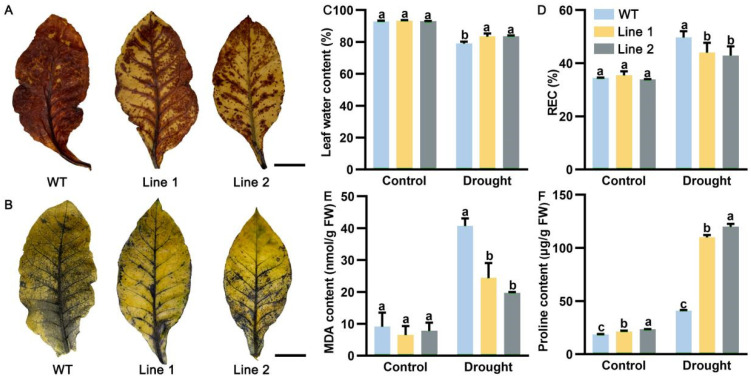
Physiological index measurement of *PoP5CS*-overexpressing *Nicotiana tabacum*. (**A**) H_2_O_2_ accumulation condition of *PoP5CS*-overexpressing *N*. *tabacum* leaves after 10 days of drought treatment. Bar = 3 cm. (**B**) O_2_^−^ accumulation condition of *PoP5CS*-overexpressing *N*. *tabacum* leaves after 10 days of drought treatment. Bar = 3 cm. (**C**) Leaf water content when *PoP5CS* was overexpressed. (**D**) REC when *PoP5CS* was overexpressed. REC, relative electrical conductivity. (**E**) MDA content when *PoP5CS* was overexpressed. MDA, malondialdehyde. (**F**) Proline content when *PoP5CS* was overexpressed. Data represent the mean ± SD of three replicates, and letters indicated significant differences by one-way ANOVA (*p* < 0.05).

To further confirm the function of *PoP5CS* in plant drought resistance, we generated two dependent *PoP5CS transgenic N. tabacum* lines, named line 1 and line 2. T2 generations of *N. tabacum* were cultivated for drought treatment. Under normal conditions, there was no significant difference between wild-type (WT) and *PoP5CS* transgenic *N. tabacum* lines (Figure 6A). Then, WT and *PoP5CS* transgenic *N. tabacum* lines were subjected to drought treatment. DNA and total RNA were extracted from WT and *PoP5CS* transgenic *N. tabacum* lines on Day 10 after drought treatment for positive transgenic plant validation. As shown in Figure 6B, when DNA was amplified using *Hygromycin* (*hygromycin* label region of pCAMBIA1301 vector)-specific primers and *PoP5CS*-specific primers, only a bright band existed in the lanes of *PoP5CS* transgenic *N. tabacum* lines. In addition, we quantified the expression level of *PoP5CS* in the above plants and found that the expression level of *PoP5CS* in *PoP5CS* transgenic *N. tabacum* lines were 29.59-fold (line 1) and 47.89-fold greater (line 2) when compared with WT (Figure 6C). After passing through 10 days of drought treatment, *PoP5CS* transgenic *N. tabacum* lines grew well in comparison to the severe drought damage condition of WT. The corresponding physiological data also matched the phenotypic observations (Figure 7). Among them, ROS were over-accumulated in WT and the water content of WT was significantly reduced compared with *PoP5CS* transgenic *N. tabacum* lines, while REC and MDA contents were significantly increased in the WT (Figure 7A–E). The above results confirmed the results that *PoP5CS* could increase drought tolerance in plants.

### 2.5. PoP5CS Regulates Drought Tolerance by Promoting Proline Accumulation

To deeper investigate the effects of *PoP5CS*, plant proline contents were determined in the control plants (*P. ostii* transformed with pTRV), *PoP5CS*-silenced *P. ostii*, WT, and *PoP5CS* transgenic *N. tabacum* (Figure 5F and Figure 7F). Under normal conditions, the proline content of the control plants (*P. ostii* transformed with pTRV) was significantly higher than the *PoP5CS*-silenced *P. ostii* lines with an average 1.95-fold difference. At the same time, the proline content of the WT was slightly lower than the *PoP5CS* transgenic *N. tabacum*. After drought treatment, proline contents were substantially increased in all of the above plants. In VIGS assay, the proline contents in the control plants (*P. ostii* transformed with pTRV) and *PoP5CS*-silenced *P. ostii* line 2 were much higher than *PoP5CS*-silenced *P. ostii* line 1. In the overexpression assay, the proline contents in *PoP5CS* transgenic *N. tabacum* lines were much higher than the WT. Obviously, the proline content was positively correlated with the drought tolerance of plants. These findings suggest that *PoP5CS* increased the drought tolerance of plants by promoting proline accumulation.

## 3. Discussion

### 3.1. PoP5CS is a Member of the P5CS Family

When plants are subjected to stress, a significant accumulation of proline occurs within their bodies, thereby enhancing their adaptive capacity to stress. As a crucial osmotic regulatory substance, proline plays a pivotal role in lowering the water potential of plant cells, strengthening their water absorption and retention capabilities, and thus safeguarding cellular osmotic balance and subcellular structural stability [29,30]. *P5CS* genes, a key role in plant proline biosynthesis, have been reported in a variety of plant species, such as *A. thaliana*, *Cajanus cajan*, *N. benthamiana*, and *Oryza sativa* [31,32,33,34,35]. In this study, we isolated the *PoP5CS* gene from *P. ostii*. The coding sequence of the *PoP5CS* gene was 1842 bp in length, encoding 613 amino acids. Liu and Wang [36] cloned the *AmP5CS* gene from *Avicennia marina*, and the coding sequence of *AmP5CS* was 2148 bp (encoded 715 amino acid protein). Yang et al. [37] isolated the *SpP5CS* gene from *Stipa purpurea*. *SpP5CS* contained a coding sequence of 2196 bp which encoded 731 amino acids. Then, a phylogenetic tree clustered PoP5CS into the branch containing *A. thaliana* AtP5CS1 and AtP5CS2. PoP5CS, *Theobroma cacao* TcP5CS, and *Gossypium hirsutum* GhP5CS were involved in multiple sequence comparison, and they had high sequence similarity. These indicated that PoP5CS is a typical P5CS family member.

### 3.2. PoP5CS Exhibits a Positive Response to Drought Stress Conditions

Exploring the expression patterns of genes under different environmental conditions can help to reveal their potential roles in cellular processes and involved metabolic pathways. *P5CS* genes play an important role in plant responses to a variety of abiotic stresses, such as drought, high temperature, and low temperature, largely determined by their expression response to different environments [32,38]. In *O. sativa*, salt-tolerant variants of plants exhibited an elevated level of *OsP5CS* mRNA transcripts under conditions of high salinity, alongside an increase in proline content, compared to their expression under standard conditions [31]. In reed canary grass, expression pattern analysis indicated an upregulation of *B231P5CS* transcripts in leaves subsequent to salt treatment (200 mM NaCl), with peak transcript abundance observed 12 h post-exposure, marking a 14.69-fold increase compared to untreated control plants [39]. In *Carica papaya*, an investigation into the expression dynamics of *CpP5CS2* under both low (7 °C)- and high (35 °C)-temperature stresses was conducted. The findings revealed that both stress conditions triggered an upregulation of *CpP5CS2* expression during storage, with this enhancement preceding the accumulation of proline [38]. In *S. purpurea*, the expression of the *SpP5CS* gene demonstrated a comprehensive responsiveness to a spectrum of stress conditions, encompassing cold, salt, and PEG stresses. Notably, a markedly significant upregulation of *SpP5CS* gene expression was observed specifically during soil drought conditions and upon subsequent rehydration treatment [37]. Here, we analyzed the dynamic expression pattern of *PoP5CS* in *P. ostii* plants subjected to drought stress. After 4 days of drought treatment, the expression of *PoP5CS* reached 1.86 times that under untreated conditions, increased to 20.61-fold at Day 8, and remarkably surged to 260.37-fold at 12 days of drought treatment. Combining the above results, *PoP5CS* strongly responded to drought stress, and this response may bring about a large accumulation of proline.

### 3.3. PoP5CS Improves Plant Drought Resistance by Accumulating Proline Content

Plants exhibit resilience against drought stress through a diverse array of intricate mechanisms, with cellular osmoregulation emerging as a pivotal mode of physiological adaptation. Proline, a pivotal osmolyte and antioxidant, assumes a paramount role in safeguarding the maintenance of cellular osmotic equilibrium and modulating the delicate balance of ROS within plant cells, thereby contributing significantly to the plant’s drought tolerance capabilities [9]. With this in mind, we attempted to validate the drought tolerance function of the *PoP5CS* gene in plants as well as to analyze the regulatory mechanisms by which they modulate drought tolerance. Through the VIGS technology and overexpression system, we obtained *PoP5CS*-silenced *P. ostii* and *PoP5CS*-overexpressing *N. tabacum* and observed their phenotypic responses to drought stress as well as changes in key physiological indicators. After drought treatment, the drought tolerance of these transgenic plants showed a significantly positive correlation with the expression levels of *PoP5CS*. The indirect assessment of cellular membrane integrity can be quantified through the REC of cellular leakages [40]. *PoP5CS*-silenced *P. ostii* had higher REC compared with the control, while exogenous spraying of proline reduced the REC of *PoP5CS*-silenced *P. ostii*. Similarly, *PoP5CS*-overexpressing *N. tabacum* demonstrated lower REC than the WT. MDA represents the degree of membrane lipid peroxidation, which indirectly reflects the degree of damage to the cell membrane system [41]. MDA accumulation in *PoP5CS*-silenced *P. ostii* leaves was significantly higher than the control, while MDA in proline-treated *PoP5CS*-silenced *P. ostii* leaves was significantly lower than that in deionized water-treated *PoP5CS*-silenced *P. ostii*. Concordantly, *PoP5CS*-overexpressing *N. tabacum* accumulated considerably less MDA relative to the WT. In addition, we assessed the abundance of ROS accumulation in transgenic plants by DAB and NBT staining and found that ROS accumulation in *PoP5CS*-silenced *P. ostii* was significantly higher than that of the control, whereas exogenous spraying of proline reduced the level of ROS accumulation in *PoP5CS*-silenced *P. ostii*. Likewise, ROS barely accumulated in *PoP5CS*-overexpressing *N. tabacum*. In a previous study, Chen et al. [42] treated *Phaseolus vulgaris PvP5CS transgenic A. thaliana* with NaCl, and found that the REC of transgenic *PvP5CS A. thaliana* was significantly lower than control plants. In *Saccharum officinarum*, the *P5CS* gene is a potential drought tolerance target gene, and overexpression of *SoP5CS* resulted in lower MDA content compared with the WT [43]. In our study, we also found that plant drought tolerance, as well as indicators of drought damage, showed a high degree of consistency with proline content, and that high proline accumulation ensured that plants maintained normal growth after drought stress, such as proline-treated *PoP5CS*-silenced *P. ostii* and *PoP5CS*-overexpressing *N. tabacum*. Similar results have been identified in multiple species, such as *S. purpurea*, *Lilium regale*, and *N. benthamiana* [33,37,44]. Our study validated, for the first time, the positive function of *PoP5CS*, a key gene for proline biosynthesis, in mediating proline-mediated drought tolerance in *P. ostii*, providing insights into the improvement of drought tolerance in *P. ostii*.

## 4. Materials and Methods

### 4.1. Plant Materials and Drought Treatment

Potting soil mixed with loam, peat, and perlite (1:1:1) was used to cultivate one-year-old plants of *P. ostii*. After three-day continuous watering, *P. ostii* plants were subjected to natural drought in a greenhouse without water supplementation. *P. ostii* leaves were collected on Days 0, 4, 8, and 12 after drought treatment. For the VIGS assay, transgenic *P. ostii* plants at the leaf expanding stage were subjected to a 15-day-drought treatment. For the heterologous overexpression assay, the T2 generation of transgenic *N. tabacum* was subjected to a 10-day drought treatment. For the subcellular localization assay, *N. benthamiana* seeds were sown in the above potting soil and incubated under long sunlight (25 °C 16 h light/22 °C 8 h dark).

### 4.2. Gene Cloning, Multiple Sequence Alignment, and Phylogenetic Analysis

We first extracted total RNA from *P. ostii* leaves and then synthesized complementary DNA (cDNA) using EasyScript RT/RI Enzyme Mix (TransGen, Beijing, China). For *PoP5CS* gene cloning, specific primers (Table S2) were designed based on reference gene sequences derived from the *P. ostii* transcriptome database (SRA: SRP161474). The 2 × Phanta Max Master DNA (Vazyme, Nanjing, China) was applied to amplify the *PoP5CS* gene, and then, purified products were ligated into the 5 × TA/Blunt-Zero Cloning Mix (Vazyme, Nanjing, China) and amplified by *Escherichia coli*. The sequence was confirmed by Tsingke (Nanjing, China). For phylogenetic analysis, the amino acid sequence of PoP5CS was used to search its homologs in other species based on the E-value algorithm in the National Center for Biotechnology Information (NCBI) database. Also, the P5CS proteins from the model plant *A. thaliana* and several woody plants (*Populus trichocarpa*, *Malus domestica*, *Pyrus × bretschneideri*, and *Vitis vinifera*) for which a full genome sequence was available were also included in the phylogenetic tree analysis. MEGA7.0 was used for protein alignment by Clustal W, and a neighbor-joining phylogenetic tree was constructed by the bootstrap method with 1000 replications, p-distance, and pairwise deletion. Sequence comparison was performed by DNAMAN 6.0.

### 4.3. RT-qPCR Analysis

Total RNA from *P. ostii* leaves (including temporally drought-treated leaves and leaves with gene silencing) and *N. tabacum* leaves with gene overexpression was extracted. Then, cDNA for RT-qPCR analysis was synthesized by 5 × EasyScript All-in-One SuperMix (TransGen, Beijing, China). NovoStart SYBR qPCR Super Mix (Novoprotein, Suzhou, China) was employed in conjunction with a BIO-RAD CFX Connect Optics Module (Bio-Rad, Hercules, CA, USA) to detect gene fluorescence signals, and the comprehensive experimental procedures can be found in Yu et al. [45]. The 2^−△△Ct^ method was employed to derive the gene relative expression level. For normalization, *P. ostii Ubiquitin* (JN699053) and *N. tabacum* tubulin alpha chain (*TUBA*, NP_001312557.2) served as internal reference controls. The primers used here can be found in Table S2.

### 4.4. Subcellular Localization

The *PoP5CS* coding sequence was amplified by gene-specific primers (forward 5′-CGGGGATCCTCTAGAGTCGACATGGCTCTTTATGATACTTTGTTCAGC-3′, reverse 5′-CACCATGGTACTAGTGTCGACCGACAACAGTGGGAGATCCTTG-3′) and then fused into a GFP tagged p35S::GFP vector; 100 ng p35S::PoP5CS-GFP, empty p35S::GFP vectors, and pCAMBIA1300-35S-NES-mCherry vectors (expressing cytoplasmic localization signals directed RPW8 2 protein) were transformed into *Agrobacterium tumefaciens*, respectively. The *A. tumefaciens* bacterial solution was resuspended in infiltration buffer (10 mM MES, 10 mM MgCl_2_, and 0.2 mM acetosyringone) and adjusted to a standard concentration (OD = 0.7). Then, the *A. tumefaciens* bacterial solution was used to infect the leaves of approximately 30-day-old *N. benthamiana*. Two days after infection, the localization regions of PoP5CS were observed by confocal laser microscopy (Nikon C2-ER, Tokyo, Japan).

### 4.5. VIGS Assay

The VIGS approach utilizing the pTRV vectors was employed to transiently silence the *PoP5CS* gene in *P. ostii*. In the TRV-based VIGS system, pTRV1 and pTRV2 are the two key viral vector components, each of which has a different function and works together to achieve the gene silencing effect [46]. pTRV2 is responsible for silencing specific genes, and pTRV1 is responsible for the replication and systematic spread of plasmids in plants. The non-conserved fragment of *PoP5CS* (from 20 bp to 338 bp) was fused into a pTRV2 vector with gene-specific primers (forward 5′-AAGGTTACCGAATTCTCTAGAGTATGGATTAGCTTTGAATGTGGATC-3′, reverse 5′-CGTGAGCTCGGTACCGGATCCTGTTCAGCCAGCTTGATGTGAC-3′); 100 ng pTRV2-*PoP5CS*, pTRV2 and pTRV1 vectors were transformed into *A. tumefaciens*, respectively. *A. tumefaciens* cultures were cultured overnight in a shaker at 28 °C to achieve an OD of 1.5. The resuspended *A. tumefaciens* bacterial solution mixtures (pTRV2-*PoP5CS* mixed with pTRV1, pTRV2 mixed with pTRV1) were kept in the dark for 1 h. *P. ostii* plants at the dormant stage with 2~3 buds were used as infestation materials. After pruning to create wounds, the roots were washed 2~3 times using deionized water. Then, the roots were immersed in the above bacterial solution, and a vacuum desiccator was employed to create negative pressure for 30 min, facilitating the penetration of the bacterial solution into the plants. Subsequent to being washed twice with deionized water, the plants were repotted into potting soil, with each experimental group comprising 15 individual plants. After a period of 30 days under cultivation, the plants had reached the leaf expansion stage. For the drought tolerance assays, the control plants (*P. ostii* transformed with pTRV) and *PoP5CS*-silenced *P. ostii* line 1 plants were treated with deionized water, and *PoP5CS*-silenced *P. ostii* line 2 was treated with proline for 3 days to test the function of proline on *P. ostii* drought resistance following approximately 15 days of drought treatment. The primers used here can be found in Table S2.

### 4.6. Heterologous Overexpression Assay

The *PoP5CS* coding sequence was amplified by gene-specific primers (forward 5′-CAGGTCGACTCTAGAGGATCCATGGCTCTTTATGATACTTTGTTCAGC-3′, reverse 5′-CGATCGGGGAAATTCGAGCTCCGACAACAGTGGGAGATCCTTG-3′) and then fused into a pCAMBIA1301 vector. The pCAMBIA1301-*PoP5CS* vector was introduced for *N. tabacum* transformation using the leaf disc method via T-DNA insertion as previously described [47]. Briefly, the pCAMBIA1301-*PoP5CS* vector was transformed into *A. tumefaciens;* the *A. tumefaciens* bacterial solution was resuspended in infiltration buffer (10 mM MES, 10 mM MgCl_2_, and 0.2 mM acetosyringone) and adjusted to a standard concentration (OD = 0.3). Then, we took leaves from *N. tabacum* sterile seedlings, infiltrated the leaves using the above mentioned bacterial solution, filtered out the solution, and co-cultivated it for 3 days in darkness. The leaves were then transferred to resistant shoot screening and differentiation medium for selection culture. The differentiated adventitious shoots were transferred to rooting screening medium for rooting culture to obtain the T0 generation. Positive *PoP5CS* transgenic *N. tabacum* was screened by antibiotic labeling, and two independent lines of *PoP5CS* transgenic *N. tabacum* (T2 generation) were used to verify their function in drought tolerance. Positive *PoP5CS* transgenic *N. tabacum* seedlings rooted in the medium were transplanted into potting soil to carry out conventional greenhouse-based cultivation. After approximately 3 months, *PoP5CS* transgenic *N. tabacum* plants with similar phenotypes were subjected to a 10-day natural drought treatment as above to verify their drought resistance function. The primers used here can be found in Table S2.

### 4.7. Physiological Index Measurement

DNA was first extracted from *PoP5CS* transgenic *P. ostii* leaves and *PoP5CS* transgenic *N. tabacum* leaves. Then, PCR and RT-qPCR were applied for positive plant validation (Table S2). Both leaves from *PoP5CS* transgenic *P. ostii* and *PoP5CS* transgenic *N. tabacum* before and after drought treatment were used for physiological index measurement. The specific experimental procedures of water content determination, REC measurement, MDA content measurement, and DAB and NBT staining were followed as previously described [48]. The proline content was measured using a reagent kit (Comin Biotechnology Co., Ltd., Suzhou, China). Briefly, 0.1 g of *PoP5CS* transgenic *P. ostii* leaves and *PoP5CS* transgenic *N. tabacum* leaves were extracted by 1 mL sulfosalicylic acid solution. After homogenization in an ice bath, the mixture was subjected to oscillation extraction at 90 °C for 10 min, followed by centrifugation at 10,000× *g* and 25 °C for 10 min, with the supernatant being collected. To 0.5 mL of the supernatant, 0.5 mL of glacial acetic acid and 0.5 mL of reaction mixture (containing ninhydrin, glacial acetic acid, and concentrated phosphoric acid solution) were added. After thorough mixing, the solution was kept in a boiling water bath for 30 min. After cooling, 1 mL of toluene was added to the test tube, followed by shaking for 30 s. The absorbance at a wavelength of 520 nm was measured to calculate the proline content.

### 4.8. Statistical Analysis

All data consisted of at least three biological replicates and error bars indicate standard error. The significant differences were analyzed by one-way ANOVA (*p* < 0.05) or by Student’s *t*-test (* *p* < 0.05; ** *p* < 0.01; *** *p* < 0.001).

## Data Availability

Data are contained within the article or Supplementary Materials.

## Supplementary Materials

The following supporting information can be downloaded at https://www.mdpi.com/article/10.3390/plants13152145/s1, Table S1: Transcriptome data of differential *P5CS* genes; Table S2: Primers used in this study.
